# Genotypic and phenotypic analyses reveal distinct population structures and ecotypes for sugar beet‐associated *Pseudomonas* in Oxford and Auckland

**DOI:** 10.1002/ece3.6334

**Published:** 2020-05-11

**Authors:** Xue‐Xian Zhang, Stephen R. Ritchie, Hao Chang, Dawn L. Arnold, Robert W. Jackson, Paul B. Rainey

**Affiliations:** ^1^ New Zealand Institute for Advanced Study Massey University Auckland New Zealand; ^2^ School of Natural and Computational Sciences Massey University Auckland New Zealand; ^3^ Faculty of Medical and Health Sciences University of Auckland Auckland New Zealand; ^4^ Centre for Research in Bioscience University of the West of England Bristol UK; ^5^ School of Biosciences and Birmingham Institute of Forest Research University of Birmingham Birmingham UK; ^6^ Department of Microbial Population Biology Max Planck Institute for Evolutionary Biology Plön Germany; ^7^ Laboratoire de Génétique de l'Evolution, Chemistry, Biology and Innovation (CBI) UMR8231 ESPCI Paris CNRS PSL Research University Paris France

**Keywords:** multilocus sequence analysis, mutation, population structure, *Pseudomonas*, recombination, sugar beet, urocanate

## Abstract

Fluorescent pseudomonads represent one of the largest groups of bacteria inhabiting the surfaces of plants, but their genetic composition *in planta* is poorly understood. Here, we examined the population structure and diversity of fluorescent pseudomonads isolated from sugar beet grown at two geographic locations (Oxford, United Kingdom and Auckland, New Zealand). To seek evidence for niche adaptation, bacteria were sampled from three types of leaves (immature, mature, and senescent) and then characterized using a combination of genotypic and phenotypic analysis. We first performed multilocus sequence analysis (MLSA) of three housekeeping genes (*gapA*, *gltA*, and *acnB*) in a total of 152 isolates (96 from Oxford, 56 from Auckland). The concatenated sequences were grouped into 81 sequence types and 22 distinct operational taxonomic units (OTUs). Significant levels of recombination were detected, particularly for the Oxford isolates (rate of recombination to mutation (r/m) = 5.23 for the whole population). Subsequent ancestral analysis performed in STRUCTURE found evidence of six ancestral populations, and their distributions significantly differed between Oxford and Auckland. Next, their ability to grow on 95 carbon sources was assessed using the Biolog™ GN2 microtiter plates. A distance matrix was generated from the raw growth data (*A*_660_) and subjected to multidimensional scaling (MDS) analysis. There was a significant correlation between substrate utilization profiles and MLSA genotypes. Both phenotypic and genotypic analyses indicated presence of a geographic structure for strains from Oxford and Auckland. Significant differences were also detected for MLSA genotypes between strains isolated from immature versus mature/senescent leaves. The fluorescent pseudomonads thus showed an ecotypic population structure, suggestive of adaptation to both geographic conditions and local plant niches.

## Graphical abstract

Fluorescent pseudomonads are the largest group of bacteria inhabiting the phyllosphere of sugar beet, but our understanding of their population structure is limited. Here, we performed multilocus sequence analysis and Biolog phenotypic assay of 152 isolates from sugar beet grown in Oxford and Auckland. Results indicate that the sugar beet‐associated pseudomonads have an ecotypic population structure, suggestive of adaptation to both geographical and local plant environments.

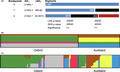

## 1 INTRODUCTION

The health and well‐being of plants is to a large extent determined by the microorganisms with which they coexist (Compant, Samad, Faist, & Sessitsch, 2019). While some bacteria can cause disease, others have the capacity to confer disease resistance and promote plant growth. A typical example concerns bacteria from the genus of *Pseudomonas*, which are ubiquitously found in soil and waters, but also form intimate associations with plants (Silby et al.,  2011). Among the ~190 *Pseudomonas* spp. validly described to date, twenty‐one are known to cause plant disease (Peix, Ramirez‐Bahena, & Velazquez, 2018). These include *P. syringae*, a well‐described pathogen for many important crops, such as kiwifruit, tomato, and beans (Arnold & Preston, 2019; Straub et al., 2018). However, certain strains of other *Pseudomonas* spp. (mostly *P. fluorescens*) possess plant growth promotion and disease suppression activities (Hsu & Micallef, 2017; Liu et al., 2018; Stritzler, Diez Tissera, Soto, & Ayub, 2018). They are capable of producing plant hormones and secondary metabolites (e.g., organic acids) that can help release nutritional substrates from the soil, particularly phosphate (Hol, Bezemer, & Biere, 2013; Oteino et al., 2015). Pseudomonads can potentially exclude pathogens that are in direct competition for available niches in the plant environments, owing to their ability to rapidly colonize plant surfaces. Additionally, pseudomonads are known to produce various antimicrobial compounds, such as cyclic lipopeptides, hydrogen cyanide, and 2,4‐diacetylphloroglucinol, which provide protection against plant infectious diseases (Flury et al., 2017; Frapolli, Defago, & Moenne‐Loccoz, 2007).

Sugar beet is commercially grown in Europe for sugar production. Early studies performed in the late 1980s with field‐grown sugar beets at the University of Oxford farm (Wytham, Oxford) showed that fluorescent pseudomonads are the largest group of bacteria inhabiting the phyllosphere, and their species composition changes during the growing season (Rainey, Bailey, & Thompson, 1994). As a representative of sugar beet‐associated pseudomonads, *P. fluorescens* SBW25 was used as a model for further genetic and biological analysis of plant–bacterial interactions (Bailey, Lilley, Thompson, Rainey, & Ellis, 1995; Rainey, 1999; Silby et al., 2009). First of all, it is interesting to note that *P. fluorescens* SBW25 is able to aggressively colonize other crops such as wheat, maize, and peas, suggesting that the interactions are not species‐specific (Humphris et al., 2005; Jaderlund, Hellman, Sundh, Bailey, & Jansson, 2008). This bacterium has, thus, likely evolved functional traits for successful plant colonization in general (Rainey, 1999). Both in vivo and in vitro studies indicated that SBW25 can protect sugar beet seedlings against damping‐off disease caused by the soilborne fungal pathogen *Pythium ultimum* (Ellis, Timms‐Wilson, & Bailey, 2000). A nonproteinogenic amino acid (l‐furannomycin) was identified as one of the antimicrobial compounds produced by SBW25 (Trippe, McPhail, Armstrong, Azevedo, & Banowetz, 2013). Furthermore, promoter trapping techniques were developed for the SBW25/sugar beet model, and their subsequent application led to identification of 139 loci whose expression is elevated during bacterial colonization in planta (Rainey, 1999; Silby et al., 2009). Some plant‐inducible genes, particularly those involved in biofilm formation and histidine utilization (*hut*), have been investigated in great detail (Gal, Preston, Massey, Spiers, & Rainey, 2003; Liu, Rainey, & Zhang, 2015). However, our understanding of the genetic diversity and population structure of fluorescent *Pseudomonas* is limited.

In a previous study, 30 fluorescent pseudomonads were isolated from the phyllosphere of field‐grown sugar beet in Oxford from where *P. fluorescens* SBW25 originated (Rainey et al., 1994). These isolates represented *Pseudomonas* present during a single growing season. They were subjected to restriction fragment length polymorphism (RFLP) analysis and phenotypic characterization of fatty acid methyl ester (FAME), biochemical properties and carbon source assimilation. The phenotypic data consistently showed that these isolates were grouped according to their time of sampling and leaf type (immature, mature, and senescent). While the RFLP data were complicated by the presence of megaplasmids, the derived genotypic groups were closely correlated with clusters generated on the basis of the phenotypic data (Tett et al., 2007). The data thus implicated niche adaptation of pseudomonads to the local plant conditions.

This initial finding prompted further analysis of the *Pseudomonas* population structure whereby a total of 108 isolates were collected in a single sampling occasion in the same field in Oxford (Haubold & Rainey, 1996). These isolates were genetically and phenotypically characterized using 10 allozyme and 23 biotype markers. The allozyme data indicated that the *Pseudomonas* population was in overall linkage disequilibrium and showed an ecotypic structure. There was a significant correlation between isolate distribution and habitat, that is, leaf type and plot. Moreover, the data also suggested a probability of frequent large‐scale recombination among certain isolates.

Multilocus sequence analysis (MLSA) has become a universal technique for studying the population genetics of bacteria, including *Pseudomonas* (Bennasar, Mulet, Lalucat, & Garcia‐Valdes, 2010; Castaneda‐Montes et al., 2018b; Ogura, Shimada, & Miyoshi‐Akiyama, 2019). It involves a comparative sequence analysis of three or more housekeeping genes, which together provide higher resolution of the phylogenetic relationships, when compared with analysis of 16S rRNA genes. Nucleotide sequences can be obtained from DNAs amplified by PCR or directly extracted from genome sequences if available. While whole‐genome sequencing (WGS) can provide information about the entire gene content, and thus an idea of the pan‐genome (Karasov, Barrett, Hershberg, & Bergelson, 2017; McCann et al., 2017), inferences on parameters governing molecular evolution and geographic structure can readily be obtained from a detailed analysis of a small set of conserved genes (Ogura et al., 2019; Straub et al., 2018). For *Pseudomonas* populations, MLSA has most frequently been used for analysis of the plant pathogenic bacterium *P. syringae* (Akira & Hemmi, 2003; Straub et al., 2018), and the opportunistic human and animal pathogen *P. aeruginosa* (Castaneda‐Montes et al., 2018b; Kidd et al., 2012). MLSA schemes have also been developed for *P. putida* and *P. fluorescens* (Andreani et al., 2014; Garrido‐Sanz et al., 2016; Ogura et al., 2019). However, MLSA has rarely been applied to plant‐associated fluorescent *Pseudomonas* (Alvarez‐Perez, de Vega, & Herrera, 2013), which comprise several phylogenetically distinct species with a common feature of pyoverdine production. Pyoverdines are siderophores secreted by fluorescent pseudomonads for iron acquisition (Zhang & Rainey, 2013). They are normally used as a marker for strain identification because of the distinguishable fluorescent yellow‐green color.

Here, we describe the population structure and diversity of fluorescent *Pseudomonas* inhabiting the phyllosphere of sugar beet (*Beta vulgaris* var. Amethyst). The same plant cultivar was grown in two geographic locations (Oxford, United Kingdom and Auckland, New Zealand), and bacterial samples were taken from three leaf types (immature, mature and senescent). We first performed MLSA analysis and obtained complete sequences of three genes (*gapA*, *gltA*, and *acnB*) for a total of 152 isolates. The MLSA data indicated that the *Pseudomonas* population was primarily associated with geographic location and leaf type from where they were isolated. We found evidence of significant recombination and identified six ancestral genotypes. Next, we performed Biolog assays to determine the ability of *Pseudomonas* to grow on 95 unique carbon sources, including histidine and its derivate urocanate. The data allowed assessment of the potential correlations between the observed genotypes and phenotypes, and a discussion of the underlying mechanisms of bacterial diversification using the dissimilation of histidine and urocanate as an example.

## 2 MATERIALS AND METHODS

### 2.1 Isolation and culture of pseudomonads

A total of 170 leaf‐colonizing *Pseudomonas* strains were isolated from nine sugar beet plants (*Beta vulgaris* var. *Amethyst*) in three 5‐m^2^ experimental plots at the University of Oxford farm, Wytham, Oxford, UK in 1993. Some of these strains (108 isolates) were subjected to a previous multilocus enzyme electrophoresis (MLEE) analysis wherein details of the sampling methods are provided (Haubold & Rainey, 1996). Briefly, three plants were randomly sampled per plot, and each plant was divided into three leaf types (immature, mature and senescent). For each leaf type, two replicate leaves were placed in a sterile plastic bag containing 5 ml sterile water. After a 1 min “massage,” the bacterial suspensions were dilution‐plated onto King's Medium B (KB) supplemented with CFC (10 μg/ml cetrimide, 10 μg/ml fucidin, and 50 μg/ml cephalosporin) from Oxoid (Hampshire, UK). After incubation at 28°C for 48 hr, two colonies were randomly picked for each leaf sample and purity of the isolates was further checked by streaking onto KB agar plates. The obtained isolates were subcultured in lysogeny broth (LB) and subsequently stored frozen at −80°C by mixing 1.0 ml culture with 0.8 ml glycerol saline solution (70% glycerol, 0.85% NaCl).

In 2004, five plants of the same *B. vulgaris* variety were grown in native soil at one site located in West Auckland, New Zealand, and *Pseudomonas* strains were isolated similarly using the KB + CFC selective plates. Isolates were coded similarly as strains from Oxford, indicating their origin of isolation: plot (1), plant (1 to 5), leaf type (1, senescent; 3, mature; 5, immature) and replicate leaf (a or b). A prefix X was assigned to distinguish the Auckland isolates from those from Oxford. For example, isolate X131b1 identifies isolate 1 from Auckland plot 1, from plant 3, from senescent (1) leaf “b.” Isolates from Oxford were additionally assigned a more simplified code from U100 to U269.

### 2.2 PCR amplification and DNA sequencing

Total DNA was extracted from bacterial cells using the CTAB‐based method as previously described (Zhang, Kosier, & Priefer, 2001). Briefly, bacteria were resuspended in 567 μl TE buffer (10 mM Tris‐HCl, 1 mM EDTA, pH 8.0) to which 30 μl of 10% SDS and 3 μl of 20 mg/ml proteinase K were added. After lysis at 37°C for 1 hr, 100 μl of 5 M NaCl and 80 μl of CTAB/NaCl solution (10% hexadecyltrimethyl ammonium bromide, 0.7 m NaCl) were added and incubated at 65°C for 10 min. DNAs were subsequently extracted with equal volume of phenol/chloroform and precipitated with 0.6 volume of isopropanol. PCR was performed in a 25 μl reaction containing 1x PCR buffer, 0.2 mM dNTPs, 1.6 mM MgCl2, 2% DMSO, 10 pmol for each primer, 30–100 ng template DNA and 0.2 units Taq polymerase (Invitrogen). After an initial denaturation at 94°C for 3 min, DNAs were amplified in 30 cycles of denaturation at 94°C for 30 s, annealing at 58°C for 30 s, elongation at 72°C for I min, followed by a 3‐min final extension at 72°C. The PCR products were purified using the Exo‐CIP Rapid PCR Cleanup Kit (New England Biolabs), before they were sent to Macrogen Inc (South Korea) for DNA sequencing.

Seven housekeeping genes were first tested in this work using available universal primers (Hwang, Morgan, Sarkar, Wang, & Guttman, 2005): *gapA*, glyceraldehyde 3‐phosphate dehydrogenase; *gltA*, citrate synthase; *acnB*, aconitate hydratase; *gyrB*, gyrase B; *coxC*, cytochrome c oxidase; *pgi*, glucose‐6‐phosphate isomerase; *rpoD*, RNA polymerase sigma factor D. Final MLSA was performed with three genes (*gapA, gltA,* and *acnB*) whose primer sequences are provided in Table 1. The three genes are almost equally distributed in the genome of *P. fluorescens* SBW25: *gapA, gltA, and acnB* are annotated to *pflu4965* (5448514–5449515), *pflu1815* (1979150–1980439), and *pflu3489* (3858403–3861012), respectively.

**TABLE 1:** Oligonucleotide primers used in this study

Primer	Sequences (5'–3')	Application
gapFSP	CGCAAYCCSGCSGAVCTGCC	Forward primer for *gapA* PCR and sequencing
gapRSP	GTGTGRTTGGCRTCGAARATCGA	Reverse primer for *gapA* PCR and sequencing
gltFSP	GAAAACTTCCTSCACATGATGTTC	Forward primer for *gltA* PCR and sequencing
gltRP	GTMCGYGCCAGGGCGAAGAT	Reverse primer for *gltA* PCR
gltRS	TAGAAGTCSACGTTCGGGTA	Reverse primer for *gltA* sequencing
acnFP	CGGTRCTSTGGTTCTTCGGCGACGAC	Forward primer for *acnB* PCR
acnRP	TTCTTCTCKACGGTCAGCAGGCC	Reverse primer for *acnB* PCR
acnFS	CCGATCTTCTAYAACACCATGGAAG	Forward primer for *acnB* sequencing
acnRS	CCAGGTCRCGCAGGGTGATGCC	Reverse primer for *acnB* sequencing

### 2.3 Assays for bacterial growth

The GN2 microtiter plates (Biolog Inc., Hayward, CA) were used following the manufacturer's instructions. Inoculants were prepared by first growing stored bacteria in LB broth and subcultured once in R2A broth (Reasoner & Geldreich, 1985). Cells were spun down and resuspended in the same volume of sterile deionized water; 100 μl of this bacterial suspension was then inoculated into 15 ml of the IF‐0 inoculating fluid from Biolog and subsequently starved at 28°C for 2 hr. Next, 150 μl of the starved cells was pipetted into each well of the GN2 MicroPlate and incubated at 28°C for 48 hr. The initial and final cell density was estimated by measuring absorbance at the wavelength of 660 nM (*A_600_*) in a Synergy 2 plate reader equipped with the Gen5 software (BioTek Instruments). Growth on histidine and urocanates was assessed with the minimal M9 salt medium as previously described (Zhang et al., 2012).

### 2.4 Data analysis

Geneious (Biomatters Ltd; Auckland, New Zealand) was used to manipulate the DNA sequences, including multiple sequence alignment, trimming, and concatenation. Unique sequence types (STs) were grouped into operational taxonomic units (OTUs) that included all STs that varied by the mean pairwise distance (0.06) of the total sample. Rarefaction analysis was performed using MOTHUR v.1.34.4 (Schloss et al., 2009). The numbers of STs and OTUs were determined by selecting the isolates at random from the population, and the procedure was repeated 1,000 times. The Simpson's index (1‐*D*) of diversity was calculated to compare variability in isolates from Oxford and Auckland, and it takes into account both the number of taxa (i.e., STs or OTUs) and the relative abundance of each taxa (Simpson, 1949). Phylogenetic trees generated in MOTHUR on the MLSA data were visualized with FigTree v1.4.4 (http://tree.bio.ed.ac.uk/software/figtree/). Statistical testing of differences in genetic diversity was performed using the permutational multivariate analysis of variance (PERMANOVA) implemented in PRIMER V6 (Anderson, 2001). Pairwise distances were used as input, and the tests were run with 9,999 permutations. Visual comparison of isolates from different ecological sources (location, plot, plant and leaf type) was performed by multidimensional scaling (MDS) using PRIMER v6. Ancestral subpopulations of *Pseudomonas* were determined using STRUCTURE software (Pritchard, Stephens, & Donnelly, 2000).

Recombination was detected using the likelihood permutation test in LDhat v2.1 (McVean, Awadalla, & Fearnhead, 2002) as previously described (Kidd et al., 2012). Evidence of recombination breakpoints was further obtained from the aligned multilocus sequences using the SBP/GARD server (Kosakovsky Pond, Posada, Gravenor, Woelk, & Frost, 2006). GARD examined 2,717 models in 00:16:25 wallclock time, at a rate of 2.76 models/second. The alignment contained 340 potential breakpoints, translating into the search space of 6,550,950 models with up to 3 breakpoints, of which 0.04% was explored by the genetic algorithm. Significance of the topological incongruence was inferred by KH test (Kishino & Hasegawa, 1989).

The Biolog growth data (*A*_660_) were analyzed by first eliminating the 28 carbon sources which did not support any bacterial growth for all tested *Pseudomonas* isolates. To cluster the growth phenotypes, a distance matrix was generated from absorbance data of the remaining 67 carbon sources using PRIMER v6. The matrix was then subjected to PERMANOVA implemented in PRIMER V6 as described above for genotypic data. Correlation between phenotype and genotype was performed using principal component analysis (PCA) and canonical variate analysis (CVA) (Renaud, Dufour, Hardouin, Ledevin, & Auffray, 2015).

## 3 RESULTS

### 3.1 Multilocus sequencing of pseudomonads isolated from the phyllosphere of sugar beet

**FIGURE 1:**
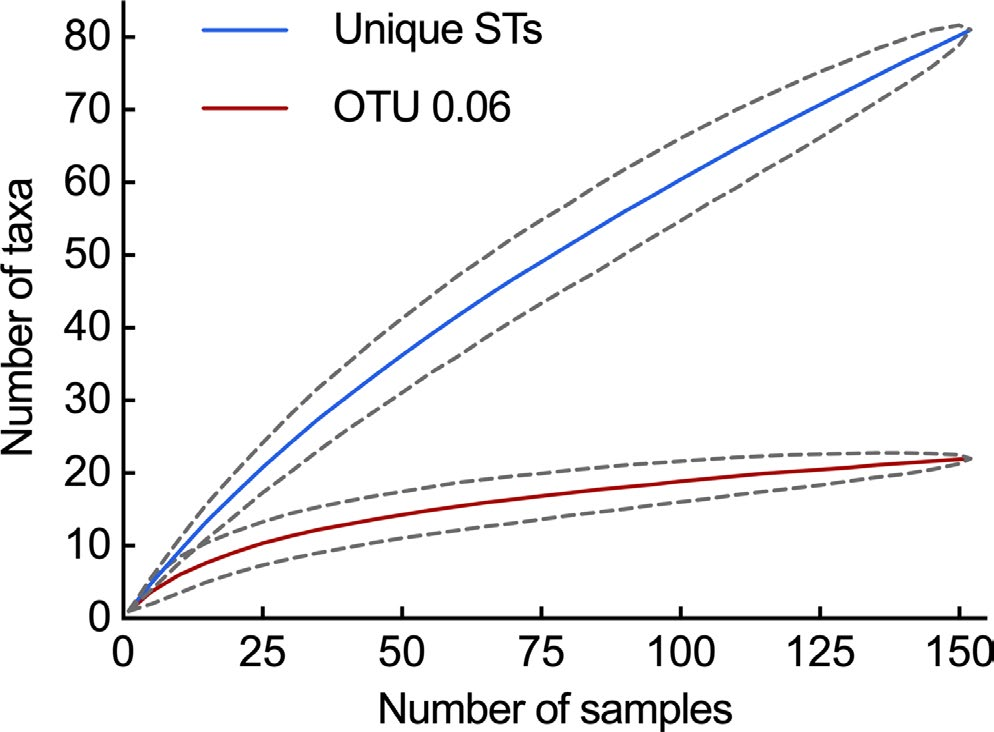
Rarefaction curves showing a near saturation of bacterial sampling. Each curve represents the mean of 1,000 replicates. Data of 95% confidence intervals are displayed with discontinuous lines

A total of 230 isolates (164 from Oxford, 66 from Auckland) were initially subjected to sequencing of seven housekeeping genes (*gapA*, *gltA*, *acnB*, *gyrB*, *coxC*, *pgi,* and *ropD*) using the universal primers (Hwang et al., 2005). However, the rates of PCR or sequencing failure were very high, suggesting that the leaf‐colonizing *Pseudomonas* populations are highly diverse. After modification of the primer sequences (Table 1), high‐quality DNA sequences were obtained for three genes (*gapA*, *gltA*, and *acnB*) in a total of 152 isolates (96 from Oxford, 56 from Auckland). The concatenated aligned sequences were 897 bp in length, and encompassed 81 unique STs and 22 distinct OTUs. Rarefaction analysis indicates near saturation of sampling at the OTU level (Figure 1a), indicating that the sample was a good representation of the genetic diversity. As summarized in Table 2, the number of segregation sites ranged from 18 to 23 for each of the three genes, and 59 identified for the concatenated sequences. Results obtained from a coalescent‐based likelihood permutation test (LPT) from LDHat (McVean, 2002) identified a recombination point within the *gapA* gene (*p* = .007), but not in the *gltA* or *acnB* genes.

**TABLE 2:** Diversity in three genetic loci in *Pseudomonas* populations

Locus	*gapA*	*gltA*	*acnB*	Concatenated
Length (bp)	303	339	255	897
Number of sequence	152	152	152	152
Polymorphism (%)	45.28	35.69	39.42	40.00
Segregation sites	23	21	18	59
Spearman's *ρ*	65.331	48.949	29.000	0.0614
Watterson's *θ*	4.134	3.774	3.235	0.0118
Rate of recombination to mutation (*r/m*)	15.8	12.9	9.0	5.22802
LPT *p*	.007	.25	.09	.08
Tajima's *D*	2.072	2.480	2.196	2.758
Fu and Li's *D*	1.830	1.777	1.686	2.357

### 3.2 Genetic diversity of the *Pseudomonas* populations

**FIGURE 2:**
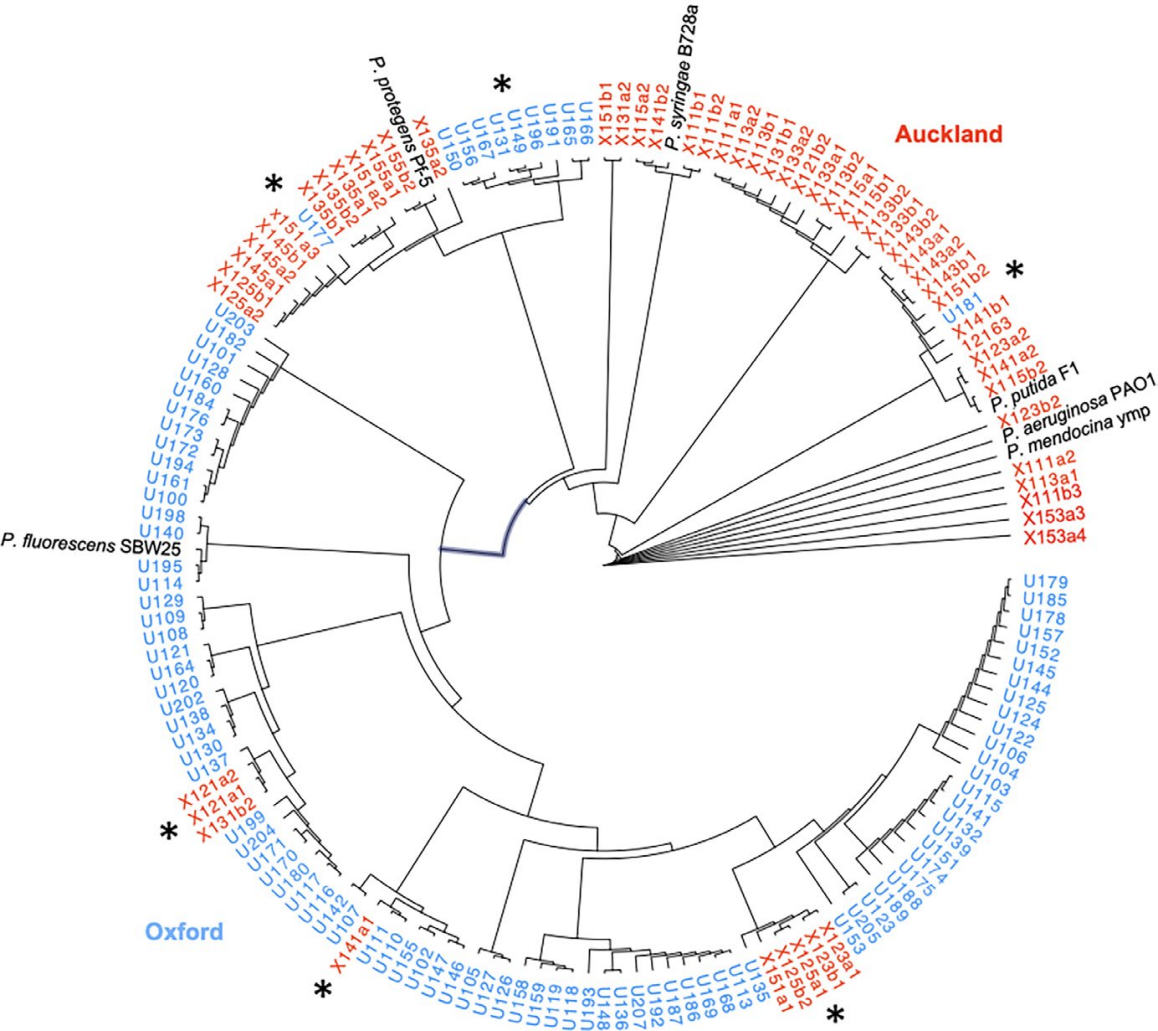
Phylogenetic relationships of sugar beet‐associated *Pseudomonas*. Isolates from Oxford and Auckland are differentiated by blue and red colors, respectively. Six reference *Pseudomonas* species were included in the analysis. Asterisks indicate the few Oxford isolates in the Auckland cluster and vice versa

**FIGURE 3:**
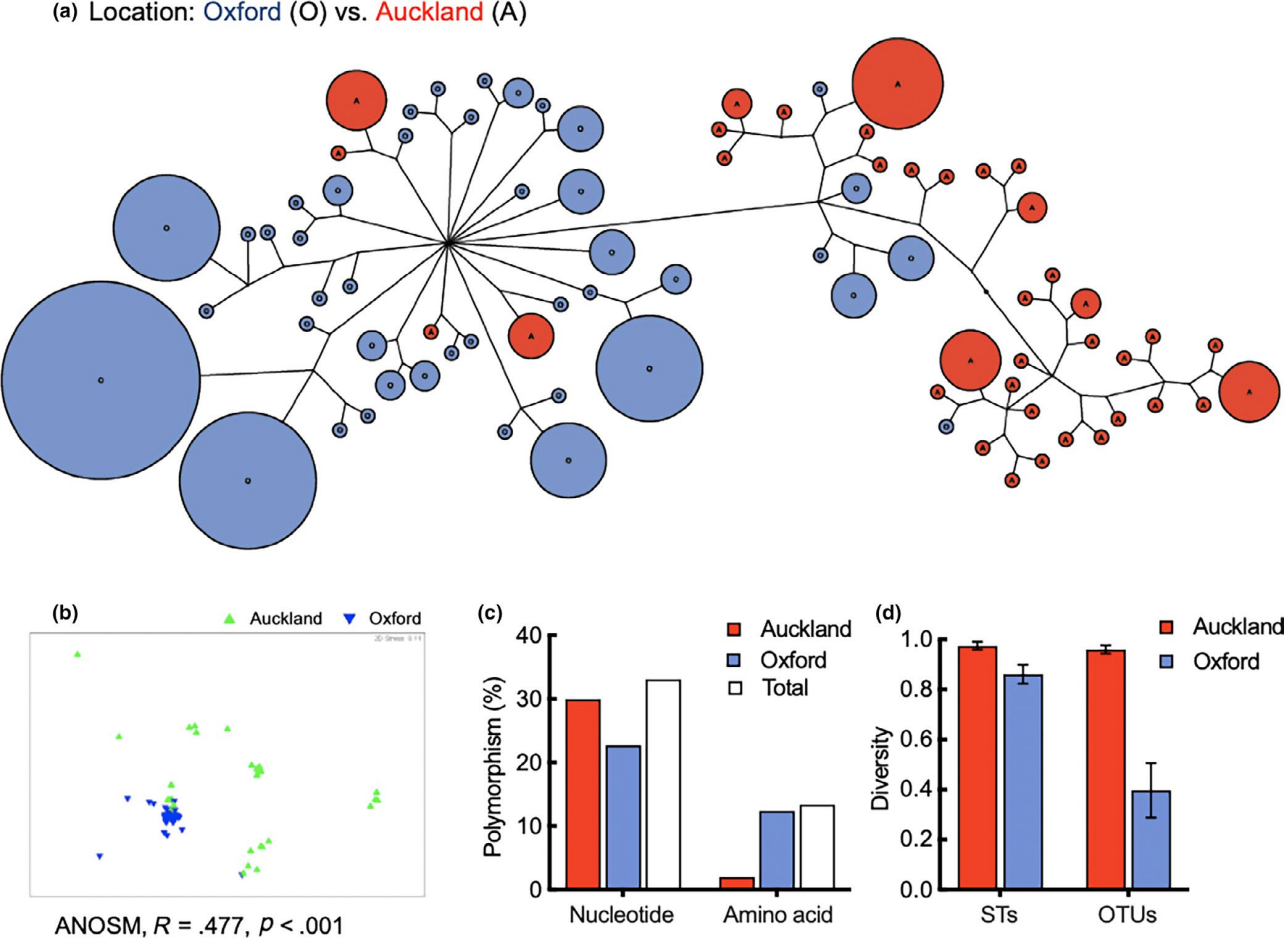
Comparative analysis of *Pseudomonas* strains isolated from Oxford and Auckland. (a) Clonal frame output displayed as an unrooted network, indicating the origination of isolation. Distance was calculated using the NJ method with Kimura 2 correction. Sizes of the circles are proportional to the number of isolates. (b) Multidimensional scaling plot showing separation of *Pseudomonas* from Oxford (blue) and Auckland (green). (c) Nucleotide and amino acid polymorphism of the concatenated sequences by location. (d) Simpson's index of diversity (1‐D) calculated on the basis of unique STs and OTUs clustered at the level of 0.06. Error bars are 95% CIs

Representative neighbor‐joining (NJ) trees are shown in Figures 2 and 3a. The *Pseudomonas* isolates were clearly separated into two major clusters according to origin of isolation, that is, Oxford or Auckland (ANOSIM in PRIMER, *r* = .477, *p* < .001 (Figure 3b); Libshuff in MOTHUR, dCXYScore 0.0254, *p* < .0001). The Oxford cluster contains the reference strain, *P. fluorescen*s SBW25, which was separately isolated from the phyllosphere of sugar beet grown in the same field. Interestingly, the Oxford cluster contains few isolates from Auckland and vice versa (highlighted by asterisk in Figure 2).

A total of 44 and 37 unique STs (81 in total) were detected in the Oxford and Auckland populations, respectively (Table 3). The two populations had no shared STs. However, when the closely related STs were grouped into OTUs at the distance level of 0.06, four shared OTUs were identified for the 11 and 15 OTUs present in the population of Oxford and Auckland, respectively (Table 3). The Oxford population displayed lower levels of nucleotide sequence polymorphism (Figure 3c) and lower diversity in terms of the Simpson's index on the basis of both unique STs and OTUs (Figure 3d). Single likelihood ancestor counting (SLAC) analysis indicated no sites under positive or negative selection for these housekeeping genes.

**TABLE 3:** Analysis of recombination in *Pseudomonas* populations by location

	Oxford	Auckland	Total population
Unique STs	44 + 0 (sharing)	37 + 0 (sharing)	81
OTUs, 0.06	7 + 4 (sharing)	11 + 4 (sharing)	22
Segregating sites	64	109	59
*d*_N_/*d*_S_	0.0219	0.0293	0.0293
Mutation rate, *θ*	0.016403	0.029109	0.01175
Recombination rate, *ρ*	high	0.031773	0.061429
Rate of recombination to mutation (*r/m*), *ρ/θ*	>10	1.09149	5.22789
Tajima's *D*	1.571	1.645	2.758
Fu and Li's *D*	1.990	2.000	2.357
LPT, *p*	.000	.025	.08
*r* ^2^	.000	.003	.260
Phi coefficient	0.4675	0.5307	0.6880
Phi, *p*	.0000	.1571	.0776

**FIGURE 4:**
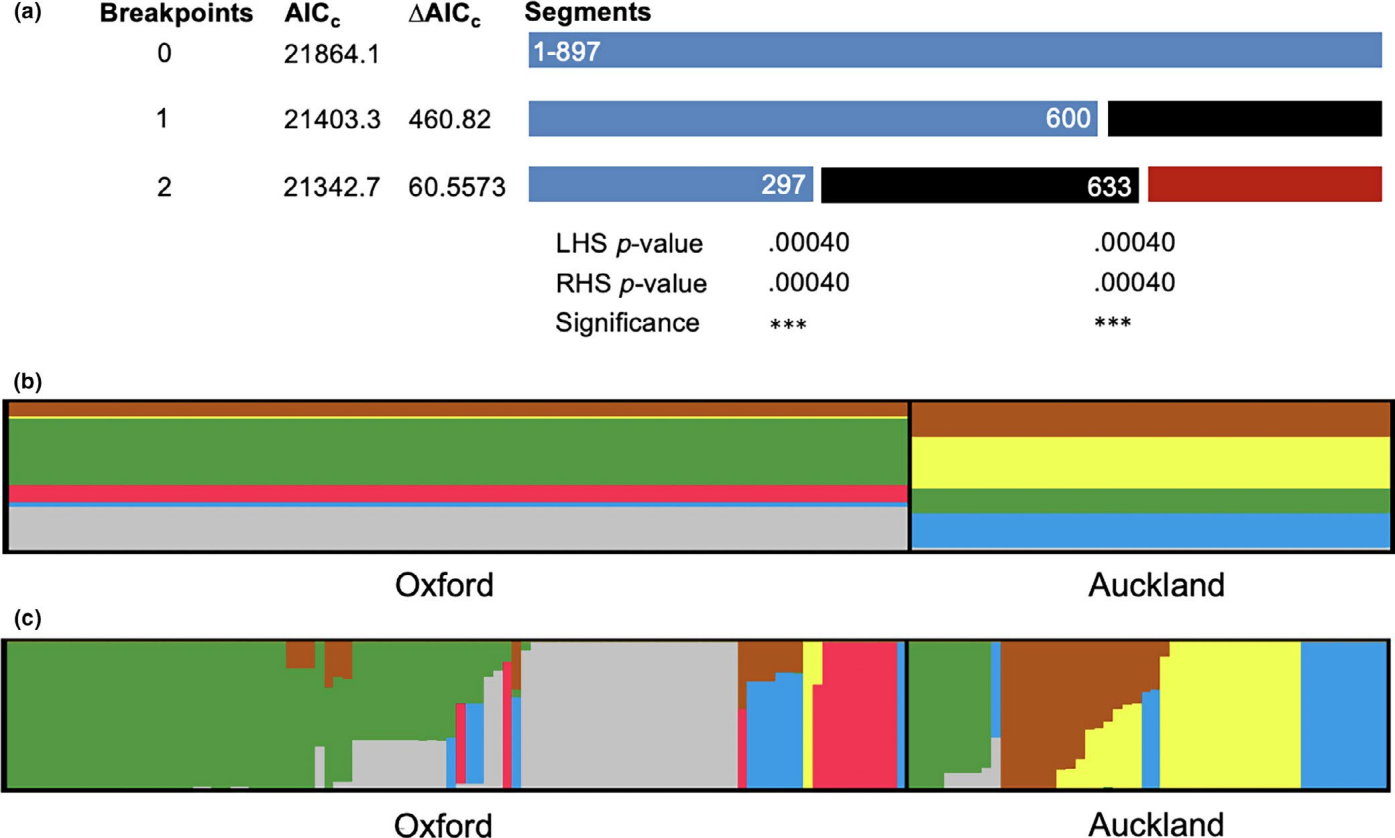
Evidence of recombination and structure of the Oxford and Auckland subpopulations. (a) Recombination breakpoints were detected using the method of GARD. Results of the Kishino–Hasegawa (KH) test are shown below the two detected breakpoints. Three asterisks indicate significance at *P* < 0.001. (b) Distribution of the six “ancestral” genotypes revealed by STRUCTURE analysis. (c) Individual isolates sharing ancestry for the Oxford and Auckland subpopulations. The ancestral genotypes are represented by different colours (b, c)

Recombination, as mentioned above, was detected in a single gene (*gapA*) but not in the concatenated sequence by the likelihood permutation test. However, when the analysis was performed with data from Oxford and Auckland separately, significant levels of recombination were noted in the concatenated sequence, particularly for the Oxford population; these were further supported by single breakpoint recombination analysis using the 10,340 model (Table 3). Further GARD analysis revealed two breakpoints with significant topological incongruence, indicating recombination breakpoints between the MLSA genes (Figure 4a).

Recombination events can be utilized to separate the isolates into populations on the basis of ancestry; that is, the presence of linked alleles in multiple isolates mandates that they have shared ancestry to some degree. Obviously, multiple sources of ancestry can be present in any one isolate. Ancestral analysis performed in STRUCTURE found evidence of six ancestral populations, and their distributions at the level of the population and isolate are presented in Figure 4b,c, respectively. Few isolates were “highly recombinant” without any predominant source of their ancestry (8% in total, mostly from Oxford).

### 3.3 Phenotypic analysis of the *Pseudomonas* populations

**FIGURE 5:**
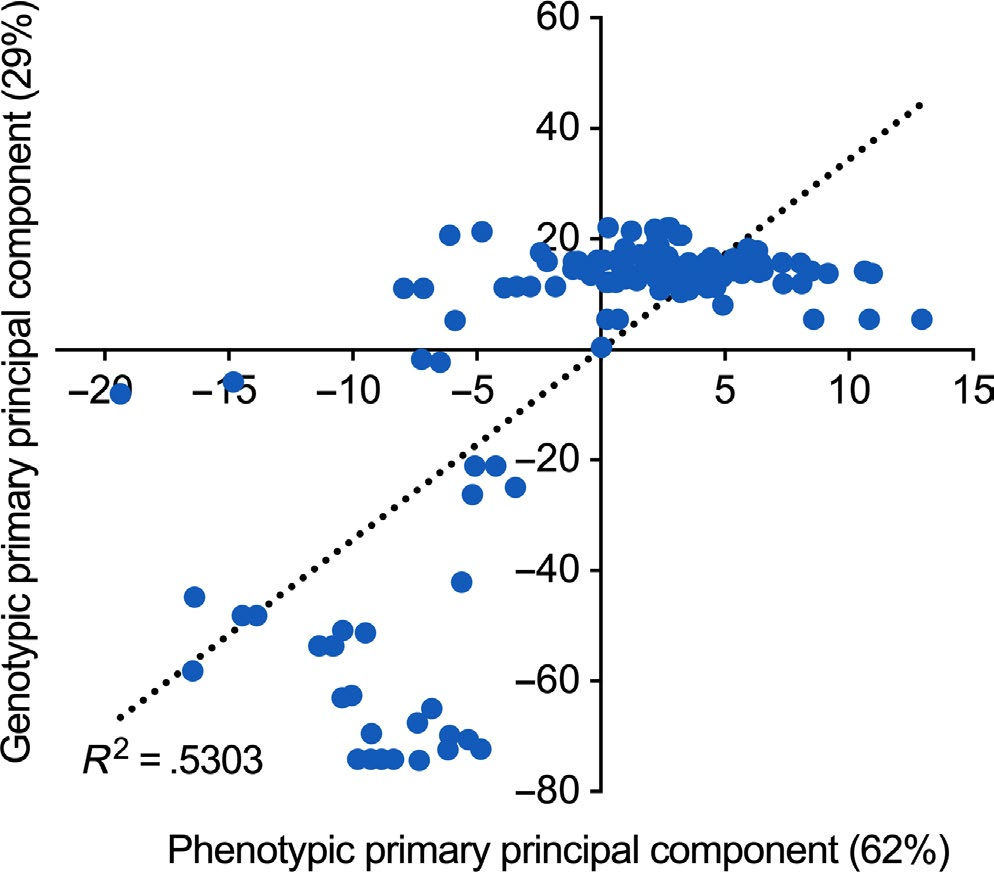
Principal component analysis showing correlations between the MLSA genotypes and the Biolog phenotypes

Growth data were obtained for a total of 95 carbon substrates included in the Biolog GN2 MicroPlate. To ensure that the phenotypic and genotypic data could be compared, the raw *A*_660_ values were used to create a distance matrix for further analysis. The results clearly indicate the presence of geographic structure, which is almost identical to the above‐described genotypic variation by location (ANOSIM, *r* = .454, *p* < *.001*). Principal component analysis (PCA) indicated that 62% of the phenotypic variability can be accounted for by the principal component, whereas only 29% of the genotypic variability can be accounted for by the principal component (Figure 5). Significantly, there was a moderate positive correlation between the phenotypic and genotypic principal components (*R* ^2^ = .5303).

**FIGURE 6:**
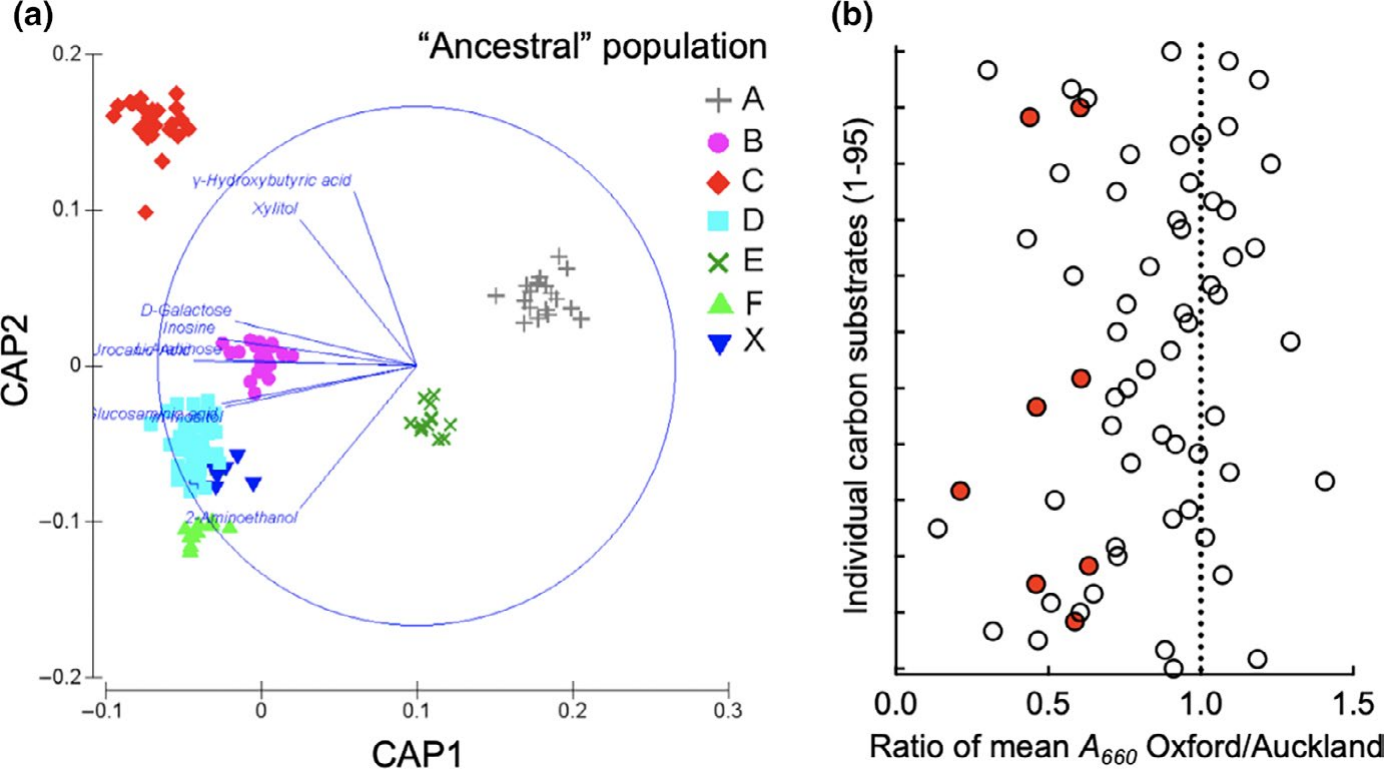
Genotypic relatedness of carbon source utilization (a) and the power of individual substrate in separating pseudomonads from Oxford and Auckland (b). (a) Canonical analysis of principal coordinates (CAP) based on a Euclidean distance similarity matrix generated from the Biolog and MLSA data. The six “ancestral” populations revealed by STRUCTURE analysis are indicated as A to F, while X denotes the other ancestral types. Only carbon substrates with *r* value larger than .8 are shown. (b) Ratio of the mean value of bacterial growth (*A*_660_) on each carbon source was calculated for isolates from Oxford and Auckland. The carbon substrates are listed in *y*‐axis in order to their location in the GN2 MicroPlate from A2 to H12 labeled from number 1 to 95. Red circles denote the eight carbon substrates of strong correlation with genotypes (Figure 6a)

Canonical variate analysis separated the *Pseudomonas* isolates into discrete populations, which matched well with the ancestral genotypes identified by STRUCTURE (Figure 6a). There was a significant correlation between carbon source utilization with the canonical axes CAP1 and CAP2 (Figure 6a). The following eight substrates had an *r* value > .8 (location in the GN2 MicroPlate shown in parenthesis): γ‐hydroxybutyric acid (D12), xylitol (C10), d‐galactose (B4), inosine (H2), urocanic acid (H1), l‐arabinose (A10), d‐glucosaminic acid (D8), and *m*‐inositol (B7).

**FIGURE 7:**
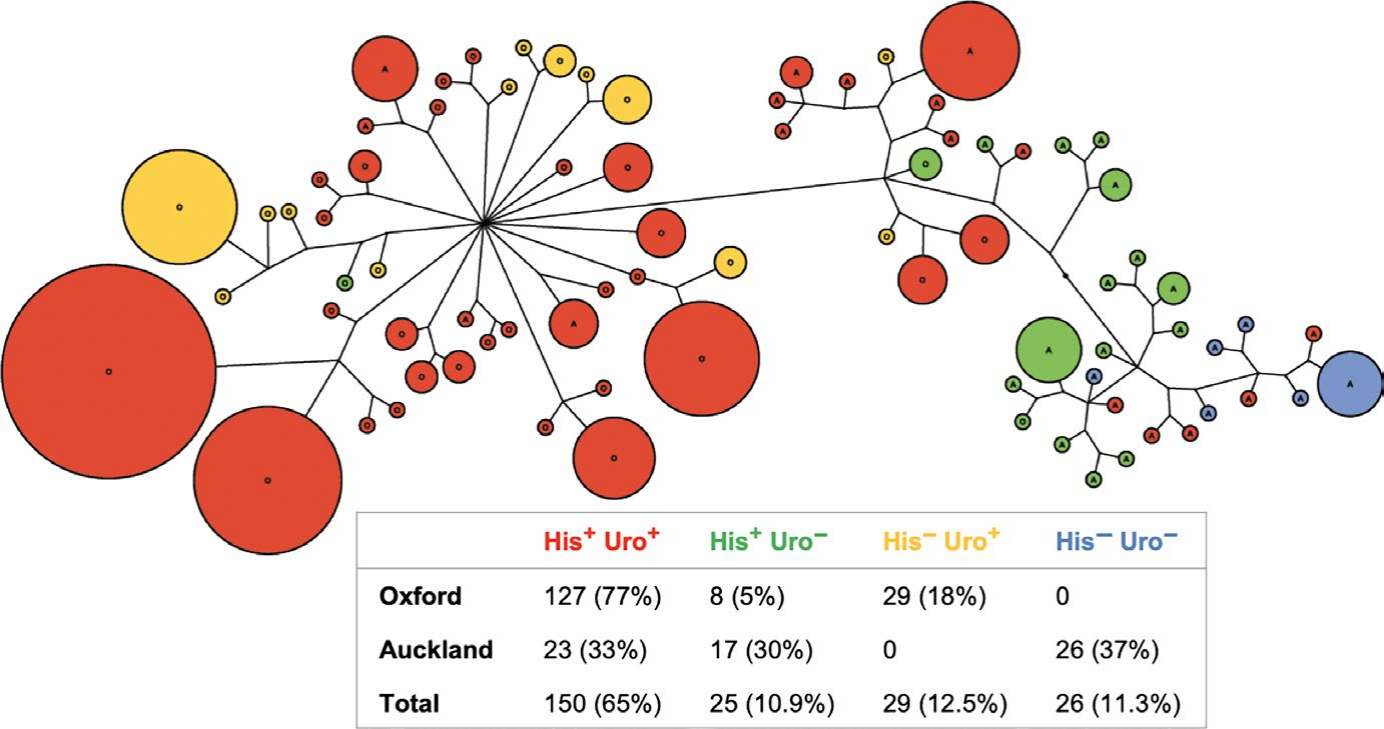
An unrooted phylogenetic tree showing the association of urocanate utilization with genotypes. The capability of bacterial growth on histidine (His) and urocanate (Uro) is marked in four different colors. The number of isolates showing the same phenotype is provided in a table below the tree, and percentage of each phenotype in the Auckland, Oxford, and total population is shown in parenthesis

To estimate the separation power of the 95 unique carbon substrates, mean values of absorbance (*A*_660_) were separately calculated for strains from Oxford and Auckland, and the resultant ratios were plotted for each substrate in Figure 6b. The data should be interpreted together with the results of canonical variate analysis (Figure 6a). Both datasets consistently suggest the power of the eight specific substrates in separating the *Pseudomonas* populations by location (Figure 6b). Of particular note are histidine (his) and urocanic acid (urocanate, uro) whose genetic basis in *Pseudomonas* has been well characterized (Zhang & Rainey, 2007). Both substrates are cocatabolized with the involvement of only one additional enzyme histidase (HutH), catalyzing the conversion from histidine and urocanate. Interestingly, there was a strong genotypic association with urocanate utilization (ANOSIM, *r* = .758, *p* < .001), but not with histidine utilization (ANOSIM, *r* = .066, *p* = .145). Further analysis indicated a clear linkage between urocanate utilization and location (location, PERMANOVA *p* = .0001; uro, PERMANOVA *p* = .0002; location × uro, PERMANOVA *p* = .0146). Figure 7 clearly shows that the Oxford and Auckland populations are well separated by their ability to grow on urocanate (His^−^, Uro^+^ vs. His^−^ Uro^−^).

Finally, it should be noted that minor but significant differences were detected between genotypes for strains isolated from immature versus mature leaves (ANOSIM, *r* = .103, *p* = .01), and also for immature versus senescent leaves (*r* = .075, *p* = .01), but not for mature versus senescent leaves (*r* = .015, *p* = .74). Similar results were found in terms of growth phenotypes on histidine and urocanate: immature versus mature leaves (*r* = .185, *p* = .01), immature versus senescent leaves (*r* = .136, *p* = .01), and mature versus senescent leaves (*r* = .05, *p* = 1.7). No significant differences were detected for the potential effects of plants and plots.

## 4 DISCUSSION

Fluorescent *Pseudomonas* are a diverse group of bacteria predominantly inhabiting the phyllosphere of sugar beet. They play an important role in determining plant health, but our understanding of their population structure is limited. Here, we present results of a MLSA analysis of fluorescent pseudomonads associated with sugar beets. The obtained MLSA data were compared with the utilization patterns of 95 unique carbon substrates. Both MLSA and Biolog analyses indicated that the sugar beet‐associated *Pseudomonas* has an ecotypic population structure with geographic location and leaf type as the most significant determining factors. Interestingly, the MLSA data revealed an unusually high recombination rate relative to mutation rate. This led to subsequent identification of six “ancestral” genotypes, which significantly differed in the Oxford and Auckland subpopulations. There was a significant correlation between the MLSA genotypes and Biolog phenotypes. Together, our results indicate that MLSA analysis with only three genes can provide an excellent basis on which to explore population structure, and a concurrent phenotypic assay can enhance our understanding of bacterial core genome evolution revealed by MLSA.

Our data consistently indicated that pseudomonads isolated from Oxford and Auckland have distinct population structures. The two subpopulations did not share any unique sequence types, and only have four common OTUs clustered according to the mean pairwise distance of the population. Given the large geographic distance between Oxford and Auckland, this finding is not surprising, as it is generally consistent with our current knowledge of microbial biogeography (Nemergut et al., 2013). The observed difference can be explained by the combined effects of historical contingencies and contemporary environmental disturbances (Martiny et al., 2006; Sun et al., 2014). More specifically, soils are a reservoir of plant‐associated microorganisms, and the species composition of microbial communities, including *Pseudomonas*, likely differ between the Oxford and Auckland sites. Of particular note is that sugar beets have been cultivated in Oxford soil for years prior to sampling, but they have never been grown in Auckland, New Zealand. Hence, one possible explanation for the observed lower level of diversity in Oxford (Figure 3d) is likely a result of long‐term selection by the sugar beet plant (Le May, Montarry, Morris, Frenkel, & Ravigne, 2019).

However, such a strong effect of geographic location in *Pseudomonas* diversification has not been reported before. Only a handful of MLSA studies are available for plant‐associated fluorescent *Pseudomonas*, but all previous reports suggested a cosmopolitan distribution, and the same genotypes were often found in geographically distant locations (Alvarez‐Perez et al., 2013; Andreani et al., 2014; Frapolli et al., 2007). For example, Frapolli, Pothier, Defago, and Moenne‐Loccoz (2012) examined a worldwide collection of plant‐colonizing fluorescent pseudomonads using MLSA of 10 housekeeping genes and 14 functional loci involved in the production of secondary antimicrobial metabolites. Their results revealed no specific linkage between genotype and geographic locations. Although the study was performed with only 30 isolates from six crops, the phenomenon was consistent with their prior work using the methods of both MLSA and PCR‐RFLP analysis (Frapolli et al., 2007; Wang et al., 2001). Therefore, further larger‐scale MLSA analyses with multiple cultivars and multiple geographic locations are necessary to verify the roles of geographic factors and local plant environmental conditions in shaping *Pseudomonas* population structure.

Recombination is a major driving force in shaping bacterial genetic diversity, but its relative importance to mutation varies greatly among different species (Gonzalez‐Torres, Rodriguez‐Mateos, Anton, & Gabaldon, 2019). A previous survey showed that the highest and lowest r/m values differed by three orders of magnitude in bacteria and archaea (Vos & Didelot, 2009). The ability of recombination to cause changes in the genome exceed that of mutation (*r/m* > 1) in more than half of the analyzed bacterial and archaeal species (56%, 27 out of 48). A significant finding in this study is the high recombination‐to‐mutation rate ratios in sugar beet‐associated *Pseudomonas*, particularly in the Oxford subpopulation. An overall *r/m* of 5.23 was detected for the concatenated sequences of three genes. This is in contrast to previous MLSA and comparative genomic studies showing that *P. aeruginosa* and *P. syringae* populations were mostly clonal, and diversity is largely determined by the process of mutation rather recombination (Castaneda‐Montes et al., 2018a; Karasov et al., 2018; Nowell, Laue, Sharp, & Green, 2016; Sarkar & Guttman, 2004; Straub et al., 2018). Relatively lower recombination levels were also reported for *P. putida* and *P. fluorescens* (Ogura et al., 2019). However, frequent recombination was detected in a MLSA study with 501 *P. aeruginosa* isolates collected from environmental, animal, and human samples in Southeast Queensland, Australia (Kidd et al., 2012). Interestingly, contrasting recombination patterns were revealed by MLSA of 38 nectar‐inhabiting pseudomonads associated with Mediterranean and South African plants (Alvarez‐Perez et al., 2013). Among the three main clades identified, two nectar groups have a mostly clonal population structure, whereas the third one showed predominant effects of recombination over mutation and exclusively consisted of isolates from floral nectar of insect‐pollinated Mediterranean plants. Given the lack of consensus marker genes for MLSA in *Pseudomonas* and the variation in strain sampling in different studies, it is difficult to understand the underlying causes for the observed higher or lower recombination‐to‐mutation rates.

Mutation and recombination are the major sources of genetic diversity, yet natural selection acts at the level of the phenotypes. A combination of phenotypic and genotypic analysis is thus necessary for the proper description of a bacterial population. Patterns of nutrient utilization are important descriptors of physiological capability, and the data can be obtained using the Biolog GN2 Microplate. This technique was developed for rapid identification of Gram‐negative bacteria through the assessment of their ability to utilize a panel of 95 different carbon sources. Data presented here revealed a significant correlation between phenotypes and genotypes defined by Biolog analysis and MLSA, respectively. Overall, the first principal component of phenotypes explained 62% of the observed diversity, but only 29% for first principal component of genotypes. Furthermore, we found that utilization of eight carbon substrates was primarily responsible for separating the Oxford and Auckland subpopulations. These included urocanic acid (or urocanate).

Urocanate is the first intermediate of the histidine degradation pathway (Zhang & Rainey, 2007). Both histidine and urocanate are included in the GN2 MicroPlate, and their utilization patterns were tested in this study. If a strain can grow on histidine (His^+^), it must have all the catabolic enzymes required for the utilization of urocanate. However, certain pseudomonads can grow on histidine, but not on urocanate (His^+^, Uro^‐^), suggesting that these strains lack a functional transport system for urocanate uptake. This led to a hypothesis that variation in histidine and urocanate utilization is attributable to genetic differences in transport systems (Zhang et al., 2012). This hypothesis was confirmed in a previous study by heterogeneous complementation after the urocanate‐specific transporter (HutTu) was identified in the model strain of *P. fluorescens* SBW25 (Zhang et al., 2012). Here, we observed a significant association of genotypes with the utilization of urocanate but not histidine. While almost all Oxford strains (95%) were capable of growing on urocanate, only one‐third could grow on urocanate in the Auckland subpopulation (Figure 7). Given the absence of historical sugar beet cultivation in the Auckland soil, our finding sits in accord with the previously proposed niche‐specific accumulation of urocanate *in planta* (Zhang, Ritchie, & Rainey, 2013). Urocanate may act not only as a nutrient but also an important signal for successful bacterial colonization. Based on this, it is logical that urocanate utilization is widespread in the Oxford subpopulation as a result of adaptation to the local conditions of sugar beet phyllosphere. Fitness improvements associated with urocanate utilization are likely to occur through changes in the uptake systems (Dean, 1995; Zhang et al., 2012). Together, our data provide an example of the genetic basis of phenotypic variation for plant‐associated fluorescent pseudomonads.

## CONFLICT OF INTEREST

The authors declare no conflict of interest.

## AUTHOR CONTRIBUTIONS

**Xue‐Xian Zhang:** Conceptualization (lead); Data curation (supporting); Formal analysis (supporting); Funding acquisition (lead); Investigation (lead); Methodology (equal); Project administration (lead); Supervision (equal); Writing‐original draft (lead); Writing‐review & editing (lead). **Stephen R. Ritchie:** Conceptualization (supporting); Data curation (supporting); Formal analysis (lead); Methodology (supporting); Writing‐original draft (supporting); Writing‐review & editing (supporting). **Hao Chang:** Data curation (lead); Formal analysis (supporting); Investigation (supporting); Methodology (supporting). **Dawn L. Arnold:** Conceptualization (supporting); Data curation (supporting); Investigation (supporting); Methodology (supporting); Writing‐review & editing (supporting). **Robert W. Jackson:** Conceptualization (supporting); Data curation (supporting); Funding acquisition (supporting); Investigation (supporting); Methodology (equal); Writing‐review & editing (supporting). **Paul B. Rainey:** Conceptualization (supporting); Data curation (supporting); Formal analysis (supporting); Funding acquisition (supporting); Methodology (supporting); Project administration (supporting); Supervision (equal); Writing‐review & editing (supporting).

## Data Availability

DNA sequences are available with GenBank Accession Numbers MT073416—MT073567 for *gapA*, MT073568—MT073719 for *gltA,* and MT073720—MT073871 for *acnB*.
